# Purified bone xenografts: A novel and efficient animal bone substitute derived from an optimized supercritical CO_2_ treatment

**DOI:** 10.1016/j.mtbio.2025.101619

**Published:** 2025-03-03

**Authors:** Solène Rota, Ludovic Sicard, Justine Perarnaud, Rémy Agniel, Raphaël Bardonnet, Catherine Chaussain, Michel Boissière, Emmanuel Pauthe, Caroline Gorin

**Affiliations:** a ERRMECe, Equipe de Recherche sur Les Relations Matrice Extracellulaire-Cellules (EA1391), Biomaterials for Health Research Group, Institut des Matériaux I-MAT (FD4122), CY Tech, CY Cergy Paris Université, Maison Internationale de la Recherche (MIR), rue Descartes, Neuville sur Oise cedex, France; b BIOBank, Bone Tissue Bank, Lieusaint, France; c Université Paris Cité, URM1333 Oral Health Inserm, Dental School, Montrouge, France; d Assistance Publique des Hôpitaux de Paris (AP-HP), Service de médecine-buccodentaire, Hôpitaux Universitaires PNVS, Paris, France

**Keywords:** Allograft, Xenograft, Bone regeneration, Supercritical fluid, Biocompatibility, Bone substitute

## Abstract

Bone xenografts represent a promising alternative to autologous or allograft transplants, yet antigenicity in animal-derived tissues remains a major limitation to their clinical use. To provide any risk of contamination or allogenic rejection, the Supercrit® process was developed to treat allogeneic human bone combining a supercritical CO_2_ treatment followed by a chemical treatment using high quantities of different solvents. The aim of this study was to produce a xenogeneic bone substitute thanks to the development of a new one-step supercritical process, 'Goxcrit', and to test it *in vivo*. This new process reduces the use of solvents by injecting them under pressure into the supercritical CO_2_ flow, while maintaining the cleaning quality of the bone matrix and better preserving its inner structure, essential for its future bone integration.

Porcine derived bone samples were treated using Goxcrit or Supercrit®, and compared with human bone treated with Supercrit®, the commercialized bone allograft. *In vitro* analyses demonstrated the absence of cytotoxicity and of the alpha-gal epitope mainly responsible for cross-species immunogenicity. Additionally, *in vivo* experiments revealed improved bone formation in rats critical calvarial defects (BV/TV and von Kossa analyses) implanted with Goxcrit samples, with bone remodeling (TRAP/ALP stains), compared to those treated with Supercrit®. These results can be attributed to the less aggressive chemical process of the Goxcrit, which preserves the bone's inner structure critical for remodeling.

Our study highlighted the interest of using a porcine bone source treated with the Goxcrit process to meet the growing demand for reliable and effective bone substitutes.

## Highlights

• One-step supercritical CO2-based treatment considerably reduces the amount of chemicals, better preserving bone bioactivity.
• Goxcrit process preserves mineral and collagen composition of the bone's inner matrix while avoiding any immunogenicity risk.
• Purified xenogeneic bone using the Goxcrit process would provide a scalable and reliable solution for bone tissue engineering.

## Graphical abstract

**Figure:**
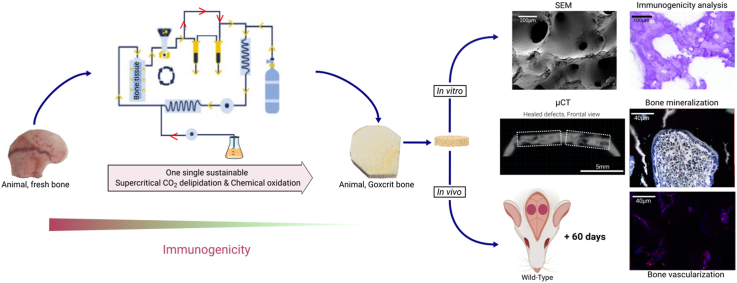

## 1 Introduction

Bone grafting is a common procedure used to reconstruct bone loss. It aims to support the formation of a well-vascularized and fully functional bone tissue following the healing process. The material must therefore have osteoinductive, osteoconductive, and osteogenic properties while minimizing any medical risks to the patient (e.g., immune reactions, infections …) [1]. A variety of grafting materials are available to surgeons, which can be classified according to their composition. These include bone from a living donor (autograft, allograft) as well as bone substitutes of animal, plant or synthetic origin.

Autologous bone grafting represents the gold standard surgical procedure due to its excellent immunocompatibility. However, large bone defects have a volume that exceeds the amount of bone available in the patient [2], leading to the development of allogenic and xenogeneic bone grafts [3,4]. Both have some drawbacks, such as potential disease transmission and immunogenicity issues due to the presence of specific-species proteins [5,6]. Allogenic bone, less concerned by immunogenicity, is primarily obtained from the replaced femoral heads of patients, which presents heterogeneous bone quality, the femoral head being often replaced in patients with osteoporosis [7]. Xenogeneic bone grafts have been developed according to the European restrictive legislation “Directive 2004/23/CE” on safety and quality of human tissues and cells [8]. They are considered as an alternative, especially since reproducibility, quantity and quality can be controlled by selecting the lineage and age of healthy standardized animals.

Bone xenografts are mainly derived from bovine, equine, porcine, or ovine sources. However, if not treated properly, they can cause a strong immunological response mainly due to the presence of alpha-1,3-galactosyltransferase protein [9]. The supply of bovine and ovine sources, susceptible to transmit prions, is becoming increasingly constrained due to the implementation of rigorous regulatory frameworks governing the manufacture of medical devices [5,10]. Due to the endorsement of quality standards by the food industry and EU regulations, swine is considered a more homogeneous and standardized source of bone tissue than equines, and is widely described [[11], [12], [13]]. To fully remove the animal proteins, the bone is exposed to a high-temperature sintering process. This technique distorts the internal structure of the bone, transforming it into inorganic hydroxyapatite-tricalcium phosphate, with the disappearance of proteins such as collagen, which ultimately hinders bone remodeling [14].

To preserve the collagen matrix and the bone structure, chemical decellularization and delipidation methods have been elaborated, albeit with a negative impact on the bone mechanical properties [[15], [16], [17]]. To overcome these effects, supercritical carbon dioxide (scCO_2_) extraction technology was developed. Beyond its critical point, scCO_2_ has both a gas-like diffusivity, which allows its easy penetration into porosities, and a liquid-like density which facilitates safe, non-toxic extraction of most organic components (e.g., adipose tissue, cells …) from bone porosities [18]. This process, already used in combination with chemical treatments to inactivate viruses, sterilize bacteria [19], and remove residual cells, has been patented (FR 2735372/US 5723012) by BIOBank industries under the name “Supercrit® process”, for the production and commercialization of allogeneic human bone tissues. However, the significant quantity of solvents used, not to mention the environmental cost, presents a real complexity in ensuring that no trace remains in the treated human bone, to enable it to be used in surgery. The use of porcine bone, known for its superior density to human bone, required a rethink of the bone treatment process to allow in-depth cleaning of the matrix without damaging it [17]. Based on an improved scCO2 protocol, the new process, Goxcrit [20], has been developed, with a potentiation of the solvent quantity/cleaning efficiency ratio compared to Supercrit®, thanks to the concomitant injection of solvents during the supercritical flow.

The aim of this study was to develop a new xenogeneic substitute for bone tissue engineering obtained with the less-aggressive Goxcrit process. Our challenge was to obtain a new Medical Device at least as safe and effective as the current marketed allogenic substitute treated with the Supercrit®. Our hypothesis was that cleaning with solvents injected under supercritical flow pressure, rather than in a passive bath, would improve quality and be less aggressive to the bone matrix. Goxcrit applied to porcine bone (aG condition) was therefore compared to the currently marketed human bone substitute (hS condition), but also to porcine bone treated with the current Supercrit® process (aS condition), in order to distinguish between the benefits of the process itself applied to porcine bone (Goxcrit vs. Supercrit®) and those of the bone source treated with the Supercrit® (human vs. porcine). *In vitro*, *in vivo* and *ex vivo* analyses were performed to assess the biocompatibility and regenerative potential of aG compared to the Supercrit® control samples (aS and hS).

## 2 Materials and methods

### 2.1 Tissue samples

#### 2.1.1 Human samples

This study was conducted in accordance with the ethical guidelines set by French bioethics laws and with a dedicated authorization delivered to BIOBank (n°FR07703T-19-01). All patients provided informed consent. Human bone tissues were collected from four living donors, three men and one woman, aged between 53 and 76 years old (61 ± 10.4 years). The samples were obtained from femoral heads collected during arthroplasty procedures.

#### 2.1.2 Animal samples

Samples were harvested from femurs and humeri, from female pigs at the end of their reproductive life, sourced from the HOLVIA abattoir (slaughterhouse) in Laval (France), which specializes in pork. All the samples were harvested from animals intended for human consumption with high quality requirements.

### 2.2 Preparation and processing of the samples

The bones were delivered to the BioBank Industry in a frozen state and were processed in the industrial facilities. Upon receipt, the bones were subjected to mechanical cleaning, which involved the removal of muscles, tendons, and cartilage using a band saw (Mado MKB-649). The epiphyses, which contain the cancellous bone, were separated from the diaphysis and then cut with a band saw into 15 mm ± 2 mm thick slices. These slices were subsequently treated either with Supercrit® or Goxcrit processes (see below section 2.2.1).

Following the treatments, cancellous bone was sectioned into discs measuring 5 mm in diameter using a drill bit (Thomas, 41PH1050STD). To cut the 1 mm in thickness, we 3D printed a specific mold for the CharlyRobot. All sample sizes were checked with a caliper and weighed to ensure a similar mass (aS: 11.7 ± 1.2; aG: 10.1 ± 1.7; hS: 10.1 ± 2.0 mg). The samples were subjected to gamma irradiation at a dose of 25 kGy to ensure sterility before experiments (cell cultures, microscopy and *in vivo* implantations).

To serve as a control in certain experiments, several bone samples were calcined at 600 °C for 6 h in a Nabertherm industrial oven, following the Supercrit® process, to retain only the mineral phase, including carbonates (Animal, calcined).

#### 2.2.1 The Supercrit® process

The Supercrit® process was developed by the BIOBank company. This process corresponds to the control group, as it has already been approved for the treatment of human bone allografts. This two-step process involved a degreasing step with scCO_2_ (pressure and temperature above 73.8 bars and 31.1 °C), and a subsequent chemical treatment at atmospheric pressure. Due to its diffusion properties and gas-like viscosity, supercritical CO_2_ dissolves the lipids contained in the bone trabeculae until saturation. The chemical treatment allows for the oxidation of residual proteins (hydrogen peroxide), viral inactivation (sodium hydroxide), and the dehydration of the bone tissue (ethanol). This process is described in detail in patent FR 2735372/US 5723012 [22].

#### 2.2.2 The Goxcrit process

The Goxcrit process (PCT/FR2023/051010) was applied to animal samples to transpose the liquid chemical treatment part of the Supercrit® process (hydrogen peroxide, ethanol) into the supercritical phase, while drastically reducing the quantity of solvent (e.g., 0.25 ml/mg vs 25 ml/mg for ethanol). After delipidation of the bones with scCO_2_, a supercritical treatment with chemical solvent additives takes place at a pressure of 160 bar and a temperature of 40 °C. Solvents are injected one after the other into the supercritical carbon dioxide stream. Each step is composed of 4 phases as described in Extended Data 1.

### 2.3 Biochemical characterization of decellularized bone

#### 2.3.1 Fourier Transform Infrared (FTIR) analysis

Cancellous bone fragments were ground using a metal mill for 5 min at a frequency of 25 s^−1^. The resulting bone powder was then mixed with potassium bromide (KBr) at a KBr/bone ratio of 300/10. The mixture was further ground to achieve maximum fineness before being compressed into a transparent pellet (1 mm thickness) using a pellet press. The obtained pellet was subsequently analyzed using a Brüker Tensor 27 spectrometer, operated via OPUS software and equipped with a mercury cadmium telluride (MCT) detector. FTIR spectra were acquired over the wavenumber range of 4000–400 cm^−1^, allowing for the characterization of both organic and inorganic components present in bone samples. All measurements were performed with a spectral resolution of 4 cm^−1^. For the inorganic matrix, the analysis focused on the characteristic absorption bands. The investigated vibrational modes included the superposition of the ν_1_ and ν_3_ phosphate vibrations (1300–900 cm^−1^), the ν_4_ phosphate vibration mode (770–480 cm^−1^), and the ν_2_ carbonate vibration mode (890–850 cm^−1^). Regarding the organic matrix, the primary absorption bands analyzed corresponded to the vibrational modes of amides: amide I (1680–1600 cm^−1^), amide II (1580–1480 cm^−1^), and amide III (1300–1200 cm^−1^). The areas under these absorption bands were integrated, and spectra normalization was applied to all considered bands.

#### 2.3.2 Thermogravimetric analysis

The analysis was carried out using a TGA Q50 (TA Instruments) on pig bones that had undergone the different processes, followed by lyophilization. The bones were ground, and 25–40 mg of each sample were analyzed. The analysis was performed under air with a heating rate of 10 °C/min. Measurements were conducted at temperatures ranging from 40 °C to 700 °C, and the data were analyzed using TA Thermal Advantage Software V5.5.24.

#### 2.3.3 Scanning electron microscopy

Samples were mounted on SEM stubs with colloidal graphite and sputtered with a 4 nm layer of platinum (EM ACE 600, Leica, Germany). They were observed using a field emission gun scanning electron microscope (SEM, GeminiSEM300, Carl Zeiss) with an acceleration voltage of 2 keV under high vacuum. Secondary electrons were collected. Scan speed and line averaging were adjusted during observation.

### 2.4 Immunohistochemical analysis: alpha-gal epitope

Fresh samples were fixed for 24 h in AFA (Carlo Erba, ref. 526263001) and then demineralized for 4 h at room temperature with RDO decalcifier (Eurobio, ref. PEURDO00-07) before being embedded in paraffin after dehydration in successive baths of ethanol, acetone, and xylene. Sections of 5 μm were prepared using a microtome and adhered to slides treated and degreased with albumin glycerin. The sections were then incubated overnight at 4 °C with the primary antibody (M86, Enzo) diluted 1:50 in 3 % BPS-BSA buffer. After inhibition of endogenous peroxidases by hydrogen peroxide, the sections were incubated with the peroxidase-coupled secondary antibody (Dako, Mouse Envision, ref. K4000). Reaction with its substrate, diaminobenzidine (DAB) (Dako, K3468), revealed the antigen-antibody complexes by the appearance of a brown label. The sections were counterstained with hemalum-eosin and mounted in aqueous medium. As a negative control, the primary antibody was replaced with 3 % PBS-BSA. The sections were observed with a Leica DM2000 light microscope, connected to a Leica DFC420C digital camera driven by LAS V4.2 image acquisition software.

### *2.5* *In vitro* experiments

#### 2.5.1 Cell culture

All cell culture media (alpha-MEM for Mouse pre-osteoblastic cells (MC3T3) and DMEM for Stem cells from human exfoliated deciduous teeth (SHED)) were supplemented with 10 % fetal calf serum and 1 % streptomycin-penicillin antibiotic. Cell passaging was performed when cells reached an 80 % confluence with 0.4 % trypsin for 5 min at 37 °C in a controlled atmosphere.

#### 2.5.2 Cell viability

The viability of SHED was assessed using the MTT assay (475989, Merck KGaA, Darmstadt). All the incubations were conducted at 37 °C in 5 % CO_2_ (n = 3 different bones/group). First, the different material conditions were incubated at four concentrations (50, 37.5, 25 and 12.5 mg mL^−1^) in triplicate in cell culture medium for 96 h. Cells (1x10^6^ cells/100 μL) were seeded in a 96-well flat-bottom microplate and incubated [21]. The supernatant was removed and replaced with the supernatant from the different incubations over a period of 24 h. The medium was then removed and 20 μL of MTT staining solution was added to each well, followed by 2-h incubation period. 100 μL of isopropanol was added to each well to dissolve the crystals. The absorbance of the viable cells was measured at 570 nm (Abs_570_) using an automated microplate reader (Infinite 200 M-Plex plate reader, Tecan). Cell viability was calculated according to the following formula:

Cellsurvival(%)=(meanODofexperimentalcompound/MeanODofnegativecontrol)x100

As mentioned in the ISO guidelines 10993-5, the viability has to be over 70 % to be considered in human application [22].

#### 2.5.3 Cell morphology

MC3T3 mouse cells were seeded at a density of 10^4^ cells.cm^−2^ without bone substitute (control condition) or in the presence of the different bone conditions. All the incubations were conducted at 37 °C in 5 % CO_2_ for 48 h. After fixation with 4 % PFA for 30 min and permeabilization with 0.1 % Triton X-100 for 30 min, samples were saturated with 1 % PBS-BSA for 1 h at room temperature. Actin filament labeling was performed using phalloidin-FITC diluted 1:50 or phalloidin-TRITC diluted 1:100. A 50 μL drop was added to each sample, followed by incubation at room temperature in the dark for 1 h. After rinsing with PBS-BSA 0.5 %, the nuclei were labeled with DAPI (ThermoFisher Scientific, ref 62248) diluted 1/1000 in PBS-BSA 0.5 % for 1 h in the dark at room temperature. Samples were stored overnight at 4 °C before confocal observation; glass coverslips were mounted with Prolong Gold mounting medium (ThermoFisher Scientific, ref P10104) and left overnight to dry in the dark. Samples were observed under a confocal microscope (CLSM - ZEISS LSM 710).

### *2.6* *In vivo* experiments

#### 2.6.1 Ethical approval and animal management

All experiments in this study were designed according to ARRIVE guidelines and performed with a protocol approved by the Animal Care Committee of the University Paris Cité (DAP #30887). Animals were maintained according to the guidelines for ethical conduct developed by the European Communities Council Directive (animal breeding agreement D92-049-01). All efforts were made to minimize their pain or discomfort. Twenty-six 10-week-old Wistar male rats were purchased from Janvier Labs (Le Genest Saint Isle, France). They were housed at stable conditions (22 °C ± 2 °C) with a 12 h dark/light cycle, enrichments and ad libitum access to water and food.

#### 2.6.2 Surgical implantation, experimental procedure and sampling

Twenty-six Wistar rats (JanvierLabs, France) were anesthetized with 80 mg/kg ketamine and 10 mg/kg xylazine hydrochloride. For each animal, the skull skin was incised, and the periosteum was peeled off to expose the bone. Two 5-mm critical size defects were made on each side of the parietal bone using a mucosa punch (Helmut Zepf 08.920.05) attached to a slow speed hand piece operating at 3000 rpm, under constant irrigation with sterile saline solution (NSK – Viva Ace) [23]. Special care was taken for the sagittal suture preservation, and minimal invasion of the dura mater. After removal of the bony discs, a cancellous bone cylinders of 1 mm thickness and 5 mm diameter was implanted in each defect. Among the 25 rats implanted, 6 received Animal Goxcrit samples, 6 received Human Supercrit® samples, 7 received Animal Supercrit® samples and 6 were left empty as the control group. Each animal received the same type of sample on each side. The periosteum was removed before wound closure to avoid any pro-osteogenic bias related to its presence on part of the defect. Wound closure was performed with absorbable suture (Vicryl Rapid 4.0, Ethicon, Johnson & Johnson). Each animal was randomly allocated per cage and per group. Immediate post-operative care included analgesia with buprenorphine (0.02 mg/kg b.w.). After surgery, rats were housed by 2 under constant conditions. Wound healing progress, material exposure or other complications were monitored daily. Body weights were monitored regularly to ensure proper feeding before and after surgery. No lethality was detected during the surgery or the post-operative period. Wound healing progressed without any sign of infection, material exposure or other complication. At day 0, 30 and 60 post surgery, rats were imaged using micro-CT as described below. Rats were euthanized and calvaria collected at day 30 and 60. The materials were fixed either in 70 % vol/vol ethanol (24 h at 4 °C) or in 4 % paraformaldehyde for further histological analyses according to the mineralization status as described below. To comply with the 3Rs, the untreated group (native calvaria) consisted of morphological data from healthy Wistar rats of the appropriate age, previously collected and regrouped in a database. In summary, for microCT analyses, at D0 and D30: aG: n = 12 defects, hS: n = 12 defects, aS: n = 14 defects, empty: n = 12 defects, at D60: aG: n = 8 defects, hS: n = 6 defects, aS: n = 10 defects, empty: n = 6 defects.

#### *2.6.3* *In vivo* micro-computed tomography (μCT)

Acquisitions were performed the day of the surgery (D0), at thirty (D30) and sixty days (D60) after surgery. After anesthesia (MINERVE Compact Anesthesia Module, 3.5 % Isofluorane under flow at 0.8 L/min), rats were imaged by X-ray micro-computed tomodensitometry (μCT Quantum FX Caliper, Lifescience, Perkin Elmer, Waltham, MA, with a source set at 90 kV, 160 μA, and acquisition with an isotropic voxel size of 60 μm). Volume reconstructions of the calvaria were performed from the DICOM files using CTvox software (Version 3.3.1, Brucker MicroCT). Before quantification, image stacks were reoriented using DataViewer (Skyscan, release 1.5.6.2, Kontich, Belgium) to the center of the defect. Then, quantification of remodeled bone was performed using a cylindrical volume of interest (VOI) of 5 mm diameter, 1 mm thick, centered on the major axis of the defect. The fraction of bone tissue on total volume (BV/TV, %), porosity (%), trabecular thickness Tb.Th (mm), trabecular number Tb.N (1 per milliliter) and trabecular connectivity (U.A.), were used to quantify and characterized newly repaired bone over the entire volume (CT analyzer software, version 1.20.8 Brucker CT), as previously described [24].

#### 2.6.4 Samples treatments

##### 2.6.4.1 Non-demineralized samples

Non demineralized samples were fixed in 70 % vol/vol ethanol (24 h at 4 °C), dehydrated in graded ethanol solutions, and embedded at −20 °C in methacrylate resin (Merck & Co., Whitehouse Station, NY) without decalcification. Resin embedded bone samples were cut (serial sagittal sections, 5 μm thickness) using Jung Polycut E microtome (Leica, Heerbrugg, Switzerland) with hard tissue blades (Leica). After immersion in a drop of 50 % vol/vol ethanol, sections were stretched to a fold-free state on polysine glass slides (Menzel-Gläser, Brunswick, Germany), covered with a polyethylene sheet, and tightly pressed on the glass slides, followed by overnight drying at RT. Deplastification was carried out in 2-methoxyethyl acetate (Carlo Erba, Val-de-Reuil, France) three times for 20 min. Rehydration of the sections was performed in graded ethanol solutions for subsequent procedures (Alizarin Red, von Kossa, Sirius red TRAP/ALP stains).

##### 2.6.4.2 Demineralized samples

Samples were fixed in 4 % paraformaldehyde for 24 h, demineralized in a pH 7.4 4.13 % EDTA solution for 3 weeks, then dehydrated in graded ethanol solutions and embedded in paraffin for sectioning to perform immunofluorescence (CD31).

#### 2.6.5 Histology, histomorphometry

Five-micrometer-thick serial sections of calvaria bone sample were deplastified, rehydrated and stained with Sirius Red, Alizarin Red, Toluidine Blue (pH 3.8), von Kossa staining or processed for ALP enzyme-histochemistry and for tartrate-resistant acid phosphatase (TRAP) revelation.

Toluidine blue staining was used to visualize connective bone matrix, and von Kossa to visualize the mineralized bone. The slides were treated for 30 min in the dark with 5 % silver nitrate (AgNO_3_) in the dark then the sections were incubated for 10 min in toluidine blue at pH 3.8. After rinsing, the sections underwent different baths of tertiary butyl alcohol then toluene.

Sirius red allows to highlight the orientation of collagen fibers. The more fibrillated the collagenous tissue, the more it can be observed with a pink color in white light, or red in polarized light. The dye binds to the collagen fibers, thereby increasing their birefringence. The deplasticized sections were stained with 1 % Sirius red, followed by counterstaining with hematoxylin.

Alkaline phosphatase (ALP) activity used as a marker of osteogenic cell differentiation. Histology sections were incubated with naphthol ASTR phosphate (Sigma–Aldrich, St. Louis, MO) and diazonium fast blue RR salt (Sigma–Aldrich) for 30 min at 37 °C (pH 9) in the presence of MgCl_2_.

Tartrate-resistant acid phosphatase (TRAP) was used as a marker of osteoclastic activity. Sections were incubated with ASTR naphthol phosphate (Sigma-Aldrich), NN-Dimethylformamide, 4-Chloro-2-methylbenzenediazonium salt (Sigma-Aldrich), sodium tartrate (Sigma S-4797) for 1 h at 37 °C, pH 5.2.

#### 2.6.6 Immunofluorescence

Samples were deparaffinized, and nonspecific peroxidases were blocked for 15 min with ortho-periodic acid. Nonspecific protein bindings were blocked with 5 % bovine serum albumin (BSA) for 1 h. Samples were incubated overnight at 4 °C, with a primary antibody against CD31 (Dako, Les Ulis, France) that recognizes endothelial cells, diluted at 1/100. Negative controls were included by the omission of primary antibodies. They were directly incubated with secondary antibodies targeting goat IgG coupled to Alexa Fluor 555. Nuclei were stained with DAPI.

#### 2.6.7 Image acquisition and quantification

Image acquisition was performed using a Lamina multilabel slide scanner (Perkin Elmer) hosted by the HistIM platform at the Institut Cochin, Paris. Slide visualization was performed with CaseViewer, 3DHISTECH's advanced slide viewing software. Images were analyzed using Fiji (Fiji Is Just ImageJ) [25], an open-source image processing package based on ImageJ (n = 12 values from 4 defects at minimum per group and per time point were used for histological quantifications)

### 2.7 Statistical analysis

Numerical variables were expressed as the mean ± standard deviation (SD). The statistical analyses were performed using Prism software version 9.0.2 (GraphPad software, La Jolla, CA). The normality of the distribution was tested with the D'Agostino Pearson omnibus normality test and the homogeneity of variance was tested with the Fisher F test. For multiple comparisons, when data was following a normal distribution with variances significantly different between groups, a Brown-Forsythe and Welch ANOVA parametric test allowing the comparison between more than two independent samples was performed. If the distribution was not following a normal distribution, a Kruskal-Wallis test was performed. For *in vivo* experiments, as two defects were performed for each animal, the bone defect was considered as the statistical unit. Differences were considered significant at p < 0.05. A Šídák's multiple comparisons correction was performed for multiple comparisons. All graphs were obtained after statistical analysis. The absence of a symbol between groups indicates non-significance (p > 0.05).

## 3 Results

### 3.1 Goxcrit treatment is as efficient as the former Supercrit® treatment

#### 3.1.1 Analysis of the bone structure after treatments

Macroscopically, no difference in the structure was detected between the different process, but the bones purified by the Supercrit® process appear whiter and those purified by the Goxcrit® process more yellow, which is the natural color of bone (Fig. 1A). In SEM, empty macropores were observed in all conditions, with identical structures (Fig. 1B a). At higher magnification, networks of fibers were visible, except for the calcined bone that exhibited an absence of organized fibers (Fig. 1B b,c). The organization of the fiber network was similar in animal-treated bone, both with Supercrit® and Goxcrit, with a more compact organization of the fibers compared to human bone. The surface of the Goxcrit bone exhibited residual fibrillary elements (Fig. 1B (b) aG condition – white arrow). To note, the collagen fibers structure of human and swine bones exhibits a high degree of similarity (Fig. S1).

**Fig. 1:**
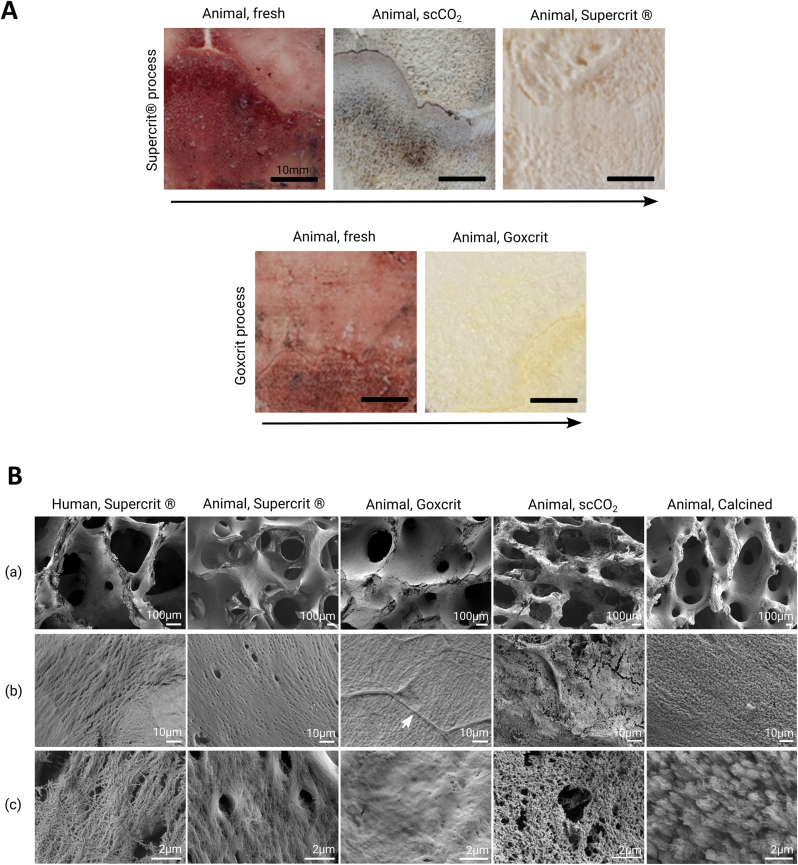
**Description of the Supercrit® and Goxcrit processes and characterization of the effects of the different processes on cancellous bone.** A: Effects of the different steps involved in the Supercrit® and Goxcrit processes on cancellous animal bone, showing the difference in the samples in terms of hue. The Supercrit® process comprises two distinct steps. The initial step is delipidation with scCO_2_, followed by chemical treatment with oxidation of residual proteins, viral inactivation, and dehydration. The Goxcrit process comprises a single step, chemical treatment in the supercritical phase. Bone treated with Supercrit® is observed to be whiter than that treated with Goxcrit, which has a yellow tint. Scale bars: 10 mm. B: Scanning electron microscopy of the cancellous bone surface following the different treatments. The Animal, Goxcrit condition exhibited a decrease in the number and size of lacunae on the surface of the material, with presence of residues (white arrows - b). Scale bars: (a) 100 μm, (b) 10 μm, (c) 2 μm. (For interpretation of the references to color in this figure legend, the reader is referred to the Web version of this article.)

##### 3.1.1.1 Degradation and conservation of the organic phase after treatments

Thermogravimetric analysis revealed that, with the exception of pre-calcined bone, all samples experienced organic loss starting at 300 °C. The mass loss was comparable between all conditions subjected to scCO_2_ treatment, suggesting that residual carbonates and proteins are present in similar proportions in these four conditions (Fig. 2A). FTIR spectra showed that bone treated with scCO_2_ retained its mineral phase as well as its amide I and II bands (1500-1700 cm^−1^), along with several peaks between 2700 and 3500 cm^−1^ (C-H and O-H), albeit with lower intensity compared to fresh bone. Following Supercrit® or Goxcrit treatments, only the characteristic peaks of the organic and mineral phases of bone were observed in the spectrum (Fig. 2B). Additional analyses, described in the Supplementary Information (SI), highlight a strong similarity in the spectra of bones treated with Goxcrit and Supercrit®. The proportions of the different components of the secondary structure organization, obtained through Gaussian deconvolution of the Amide I band shown similarity between all the treated samples (Suppl. Table S1). Indeed, the area ratios (integration) of the Phosphate, Amide I, and CO_3_ bands are listed in Suppl. Table S2. Finally, the analysis of the ν_4_PO_4_ band, located between 500 and 700 cm^−1^, provides insight into the crystallinity index of the bone mineral phase (hydroxyapatite) and further confirms the strong similarity between the profiles of bones treated with Supercrit® and Goxcrit (Suppl. Table S3).

**Fig. 2:**
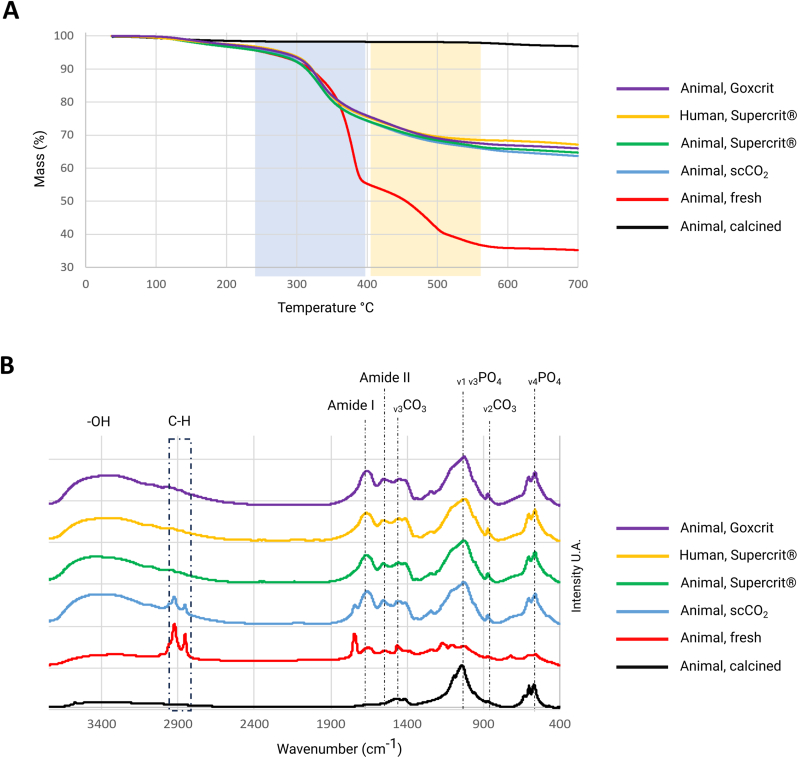
**Quantitative analysis of cancellous bone composition after Supercrit® and Goxcrit processes**. A: Representative thermogravimetric analyses under air with a 10 °C/min ramp of different conditions of treatment on bone, with quantitative results, showing a similar pattern of degradation in bones treated with a process using scCO_2_, and a preservation of organic compounds compared to the calcined bone. Fresh bone has a higher mass loss due to the presence of all its constituents (no treatment), and the characteristic bands of the organic or mineral phase are therefore difficult to observe due to the presence of all its constituents. FTIR analysis confirmed the absence of an organic phase in the calcined bone with the absence of the amide bands (1500 -1700 cm^−1^), and only the carbonate and phosphate peaks present. Freeze-dried samples B: Representative Fourier transform infrared spectroscopy (FTIR) Spectra of different conditions of treatment on bone confirming the results obtained with thermogravimetric analysis, showing a preservation of C-H, Amide I and Amide II compounds in bones treated with scCO_2_, Supercrit® and Goxcrit processes, in comparison to calcined bone.

#### 3.1.2 Immunogenicity of process-treated animal bones

Immunogenicity and residual cellularity of porcine bones following treatments was conducted using alpha-gal antigen labelling by immunohistochemistry and hemalum-eosin counterstaining (Fig. 3A). In histological sections of the “Animal, fresh bone” condition (Positive control), the presence of bone marrow cells could be observed, and was still present in the “Animal, scCO_2_” condition. The specifically labeled cells were notably less found than in the “Animal, Fresh” condition with a clear preference for localization at the junction between adipocytes (black arrow). Bone marrow was absent in the Supercrit® and Goxcrit samples (hS, aS and aG) and no tissue residues were found in the bone trabeculae.

**Fig. 3:**
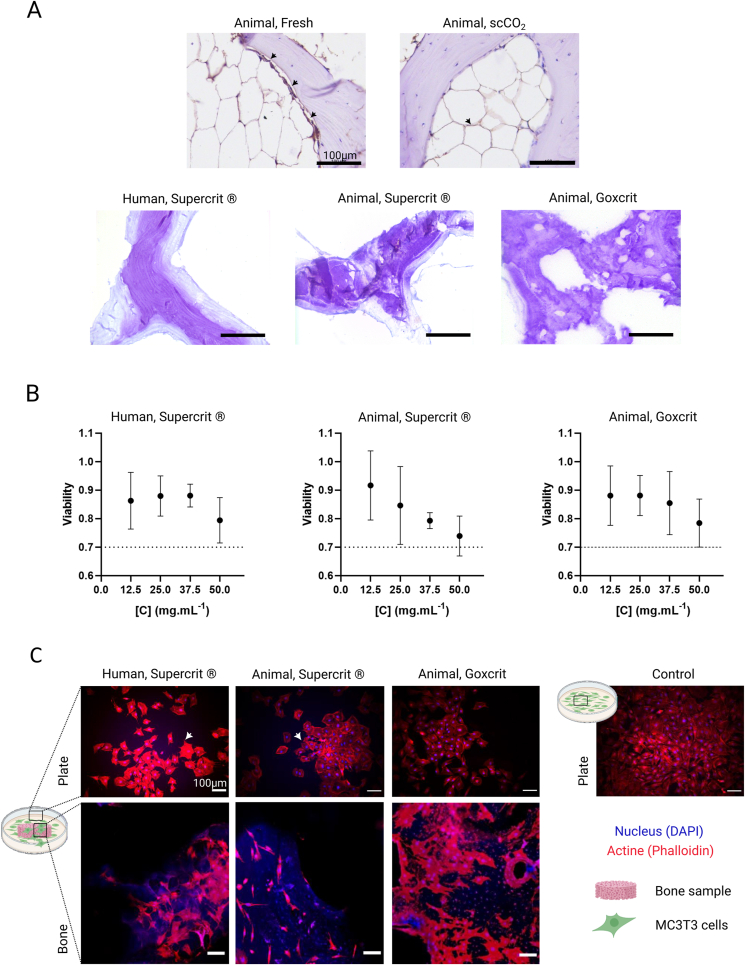
**Immunologic and *in vitro* characterization of the bone samples after Supercrit® and Goxcrit treatments.** A: Anti-alpha gal immunostaining of the bone samples after the different treatments, with hemalum-eosin counterstaining (anti-alpha gal labels: black arrow). The presence of specific cell labeling (indicated by the arrows) was clearly distinguishable in cell clusters located between adipocytes and along the trabecular bone in fresh and scCO_2_ conditions. Specific anti-alpha gal labeling was not visible in the Supercrit® and Goxcrit conditions. Scale bars: 100 μm. B: Cell cytotoxicity as a function of a gradient of extracts (n = 3 different bone samples/condition/concentration). MTT assay conducted in accordance with the standard protocol 10993-5. No statistically significant difference between the Supercrit® and the Goxcrit process. C: Confocal microscopy of the surface of different bone samples after 48 h of MC3T3 cell culture revealing the presence of actin cytoskeletons that were spread out, as well as membrane extensions and filipodia (Actine/Phaloidin in red, Nucleus/DAPI in blue). Scale bars: 100 μm. (For interpretation of the references to color in this figure legend, the reader is referred to the Web version of this article.)

#### 3.1.3 Bone treatments preserved cell viability

The MTT assay was performed to assess cell survival in contact with supernatant obtained from the different conditions (hS, aS and aG). Our objective was to assess the potential cytotoxicity of the new protocol Goxcrit in comparison with the well-established protocol Supercrit® already used to treat marketed human bone substitutes. In all groups, cell viability ratio was over 0.7, the threshold for ensuring non-cytotoxicity. No statistically significant differences were found between the groups regardless of the bone source (animal vs human) or treatment process used (Supercrit® vs Goxcrit) (Fig. 3B).

#### 3.1.4 Cell morphology is similar to adherent cells

A cell morphology assay was performed to assess whether osteoblast lineage cells (MC3T3) could develop on bone treated with the Goxcrit protocol. At 48 h, no difference in the cell's morphology was observed between the 3 groups (hS, aS and aG) whether on the cultures in direct contact with the treated bones or with their supernatant obtained from the different conditions (hS, aS and aG). In addition, all cells appeared spread out, with membrane extensions characteristic of their phenotypic adhesion profile (white arrows) (Fig. 3C).

### 3.2 Xenogeneic bone implant treated by Goxcrit enhance bone remodeling *in vivo*

Bone samples (5 mm-diameter, 1 mm-thick) (Fig. S3) were implanted in the rats calvaria critical bone defects and monitored over 2 months to follow-up the bone regeneration regardless the two bone sources (human or porcine) or their treatment processes (Supercrit® or Goxcrit).

#### 3.2.1 Evaluation of bone reparation by μCT-imaging

Three-dimensional reconstruction of the rat skulls showed complete coverage of the surface defect at D60 only in the aG condition. Indeed, on coronal sections, the material was filled by a mineralized substrate, whereas in the other implanted materials the bone trabeculae were still visible (Fig. 4A). To note, the hS sample structure exhibited a greater degree of macroporosity than that observed in the two other groups (aS and aG), due to the source of bone, the human bone being less dense than the animal one, explaining the significant lower BV/TV of hS, and the difference in the connectivity at D0 (Fig. 4B and C). Indeed, the size of the medullary cavities was larger for samples of human origin (hS) with fewer but thicker trabeculae (Fig. S4). Surprisingly, no significant difference in BV/TV was observed over time within the hS and aS groups. The BV/TV was only significatively increase in the aG groups at D30 and D60 compared to D0. The aG group showed a significantly higher BV/TV at D30 and D60 compared to all the other groups. To note, the mean BV/TV at D60 in the aG condition was higher than the reference value of the native calvaria (19 ± 6 %), as the implanted material was 1 mm-thick and the mean native calvaria thickness was around 0.5 mm-thick (Fig. 4B).

**Fig. 4:**
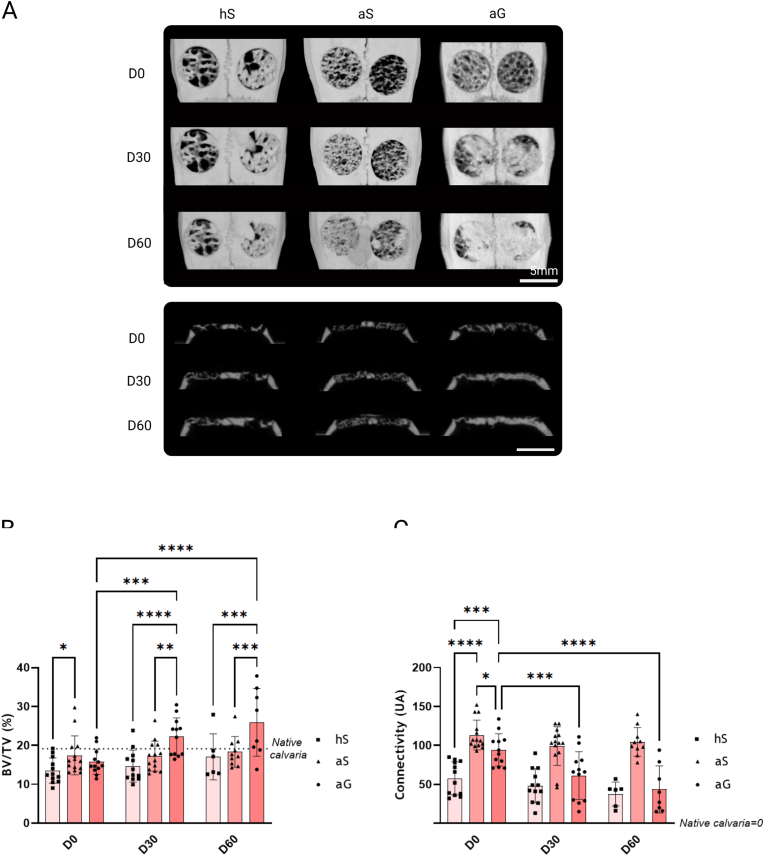
**Comparison of bone repair in the different conditions.** A: Representative and three-dimensional and coronal images of bone defects created in rats calvaria, in three conditions: human-derived bone sample treated with Supercrit® (hS), animal-derived bone samples treated with Supercrit® (aS) and by Goxcrit (aG), after 0, 30 and 60 days, revealing nearly complete filling in aG condition at 60 days. B–C: BV/TV (B) and Connectivity (C) evolution at 0, 30 and 60 days, showing a significant difference between the aG condition and the two others, either in the BV/TV or the connectivity [D0 and D30: aG: n = 12 defects, hS: n = 12 defects, aS: n = 14 defects, empty: n = 12 defects, at D60: aG: n = 8 defects, hS: n = 6 defects, aS: n = 10 defects, empty: n = 6 defects]. The connectivity index represents the interconnectivity of the cancellous bone lacunae. A reduction decrease in the connectivity index should indicate the remodeling of the implanted cancellous bone material into a cortical bone structure, the greater the number of connections, the higher the index is. No significant reduction in the connectivity of the hS and aS group was measured. ∗p < 0.5, ∗∗p < 0.01, ∗∗∗p < 0.001, ∗∗∗∗p < 0.0001.

To evaluate the remodeling process of our implanted bone**,** the connectivity index was measured. Only the aG condition showed a significant decrease of its connectivity overtime, with a halving of the connectivity at D60, indicating the transition from cancellous to cortical bone (i.e., connectivity = 0) (Fig. 4C).

#### 3.2.2 Histological analysis of bone remodeling

To discriminate more precisely the mineralized tissue over time inside each defect, von Kossa/Toluidin blue staining was performed (Fig. 5A – red frames). aG condition exhibited at an early stage (D30) an osteoid matrix with bordering osteoblasts and osteocytes within lacunae which are indicative of functional bone. In contrast, the other groups exhibited these characteristics only at a later stage (D60) (Fig. 5B). von Kossa quantification was also consistent with BV/TV analysis with higher mineralized tissue measured in the aG condition. The only difference encountered compared with the CT analysis was for the hS condition with a significant increase in mineralized tissue between D30 and D60.

**Fig. 5:**
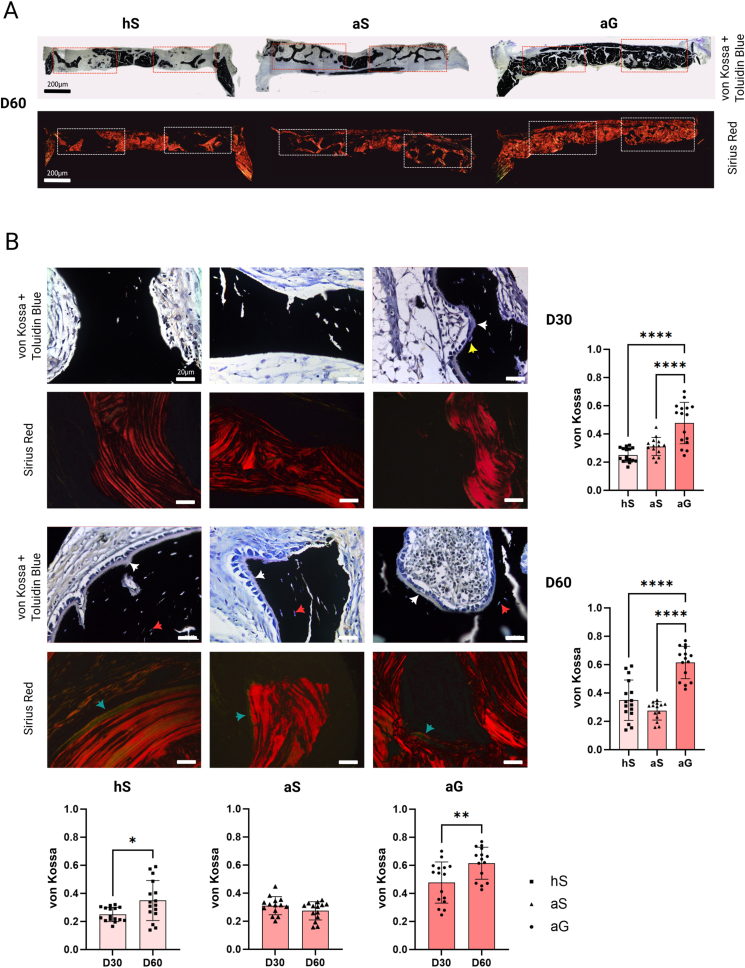
**Comparison of the mineralizing process in the different conditions.** A: von Kossa and Sirius red staining of representative coronal sections of rat calvaria at 60 days with the defects enhanced (rectangles), in the three conditions: human-derived bone sample treated with Supercrit® (hS), animal-derived bone samples treated with Supercrit® (aS) and by Goxcrit (aG). Scale bars: 200 μm. B: von Kossa and Sirius red staining with a focus on the trabeculae. A mineralized matrix (shown in black) including osteocytes within lacunae (red arrows) was surrounded by an osteoid tissue (white arrows) with bordering osteoblasts, some being included (yellow arrows). At D60, in all the conditions, the mineralized tissue exhibited histological characteristics comparable to functional bone, unlike aG, which shows off its features as early as D30. Sirius red enhanced the fibrillar collagen organization and remodeling. Quantitative analysis [hS n = 16, aS n = 14, aG n = 15 slices over 4 defects/group/time point] revealed a significant evolution in the aG condition compared to the two other conditions (hS and aS). Scale bars 20μm. ∗p < 0.5, ∗∗p < 0.01, ∗∗∗∗p < 0.0001. (For interpretation of the references to color in this figure legend, the reader is referred to the Web version of this article.)

The arrangement of the fibrillar collagen was assessed by Sirius red staining. In a non-remodeling bone, as for the different bone samples, the collagen is organized in layers of fibrillar collagen (red in polarized light) with interposition of non-fibrillar collagen (black in polarized light) (Fig. S5). Under repair process, these well-organized layers tend to disappear to make way for clusters of fibrillar collagen (i.e., green and yellow fibers differentiating the various states of organization). This was observed earlier at D30 in the aG condition and was found in all conditions at D60 (Green arrows, Fig. 5B).

**Fig. 6:**
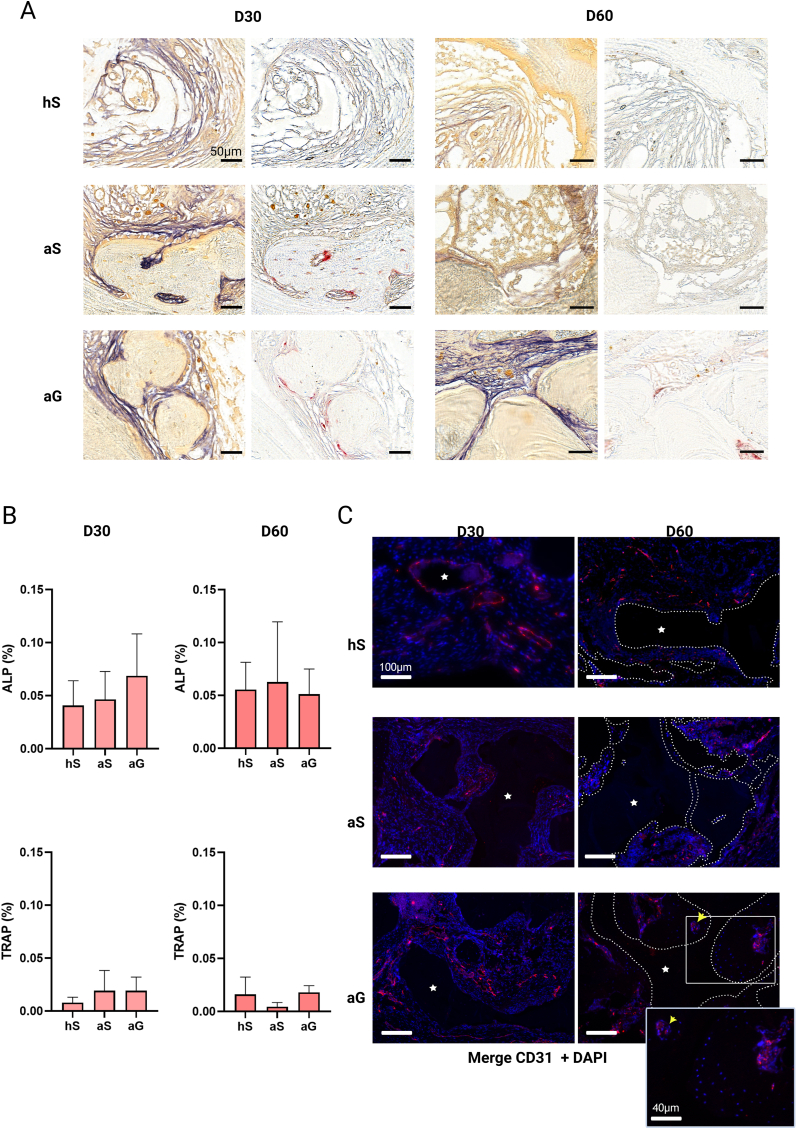
**Bone remodeling activity and vascularization.** A: ALP and TRAP staining of representative histological sections at 30 and 60 days (scale bar: 50 μm), showing the presence of a functional bone with osteoclast activity (TRAP, red staining) and osteoblast activity (ALP, purple staining). B: Quantitative assessment of TRAP and ALP activities [n = 12 values from 4 defects per group and per time point] showing no significant difference between groups. No difference was found between groups over time (data not shown). C: anti-CD31 immunofluorescence of the bone samples after the different treatments, at 30 and 60 days, with presence of vessels surrounded by bone (yellow arrow) and included cells (insight, scale bar 40 μm). CD31: Red, DAPI, Blue. Scale bars 100 μm. (For interpretation of the references to color in this figure legend, the reader is referred to the Web version of this article.)

This active bone remodeling was also shown with osteoblastic cell activity (ALP activity - purple staining bordering the mineralized tissue) and osteoclastic cell activity (Trap activity – red staining in contact with the mineralized tissue), without any difference between the groups (Fig. 6A and B). The balance being in favour of osteoblastic activity.

**Extended Data 1:**
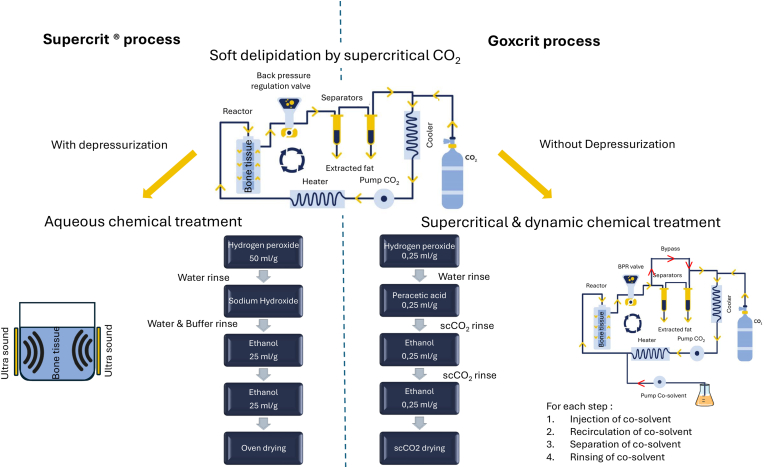
**Diagram of the Supercrit® and Goxcrit processes, with proportion of solvent used, emphasing their drastic reduction with the Goxcrit process.** For the Goxcrit process, each step is composed of 4 phases: 1) injection of the solvent into the scCO_2_, 2) recirculation of the solvent in the system to increase the contact time with the samples, 3) a separation phase to collect and eliminate the solvent to be able to inject the next one, and 4) a scCO_2_ rinsing phase between each use of solvent to eliminate the residual one.

In addition, in all conditions, vessels (CD31-labeled) were found in trabeculae delimited by non-remodeled bone at D30 (Fig. 6C - white stars), and only aG showed vessels fully embedded in a newly formed bone with internalized cells at D60, indicating an active process (Fig. 6C - yellow arrow top right).

## 4 Discussion

This study showed that the new substitute ‘porcin bone treated by the Goxcrit process (aG)’ had significantly higher bone repair capacity after implantation *in vivo* than the ‘porcine bone treated with the patented Supercrit® process (aS)’. Although both processes are based on supercritical CO_2_ technology for bone treatment, the differences in their implementation had an impact on bone regeneration, the aG group responding better than the aS group. This study can therefore be considered as a non-inferiority trial compared with the current marketed hS substitutes, whose efficacy is well-known. However, it was astonishing to see that the aG substitutes showed better results than the already marketed hS bone substitutes, which may lead to further studies in the future.

Nevertheless, the choice of the porcine source was carefully considered in order to provide greater certainty with regard to its potential natural immunogenicity in humans. Indeed, in aged swine, bone marrow exhibits a reduction in cellular diversity, resulting in lower expression levels of biological markers (unpublished data). This may reduce the need for aggressive treatment to achieve a biocompatibility similar to that of allograft. Thus, only aged swine were selected as the sources of bone samples.

scCO_2_ is a particular state of carbon dioxide, which is reached when the critical pressure (73.8 bar) and the critical temperature (31.1 °C) are exceeded. Under these conditions, CO_2_ combines a high solvent power and a very low viscosity. The chemical nature of CO_2_ allows solubilization of apolar and thermosensitive compounds. This property places it as a very good candidate to extract the lipids and treat the mineralized matrix by entering through the pores and micropores of the bone tissue [18,26]. Moreover, the low working temperature (Tc = 31 °C) preserves the organic constituents of the matrix [27]. scCO_2_ in the supercritical state also has viro-inactivating properties, essential to allow their implantation [19], but not sufficient to meet the ANSM's (French National Agency for Medicines and Health Products Safety) requirements concerning the prevention of risks related to the transmission of viruses and prions. To overcome this issue, chemical treatments to secure the graft are necessary [8,28].

The Supercrit® process is a combination of a degreasing step with supercritical fluids followed by a chemical treatment in an aqueous phase at atmospheric pressure. This last step contains a chemical oxidation of the residual proteins (hydrogen peroxide), a viro-inactivation of the matrix (sodium hydroxide) and a dehydration (ethanol). This combination has shown to have a sterilizing effect on bacteria, spores, and certain viruses [29]. This process has been designed to preserve the collagenous structure of the bone while meeting the safety criteria required for a graft material.

The scCO_2_ process is totally neutral on the mineral and collagen composition of the bone matrix, preserving the integrity of the trabecular bone tissue and a mechanical strength comparable to fresh bone [30]. In order to proceed with the characterizations, it would be interesting to study the mechanical properties of bone as a function of its origin (human versus porcine) and processing (Supercrit® versus Goxcrit), in order to draw sound conclusions about the effect of these two processes on the mechanical properties of these bone substitutes, and the feasibility of their use in a mechanically stressed bone [17,27,31]. Thus, the osteoconductive properties of supercritical CO_2_ processed bone were comparable to autogenic bone. Nevertheless, the chemical treatment used (described above) have a deteriorating effect on matrix proteins.

Indeed, several articles highlight the deleterious effects of hydrogen peroxide on the protein structure of bone, but also on its mechanical properties. Dumas et al. observed a slight decrease in cell proliferation and a decrease in ALP activity after one week of culture on bone matrices treated with hydrogen peroxide and sodium hydroxide (separately or combined), but not with ethanol [32]. When comparing our SEM results with those of Dumas et al. the same fibrillar structure is observed for samples treated with a sodium and hydrogen peroxide solution. Although this fibrillar structure is described as altered by Dumas et al., the samples treated by Supercrit® remained organized and better preserved than calcined bone [32]. Indeed, Sirius red staining showed that the fibrillar network structure corresponded to a well-organized collagen network despite the amount of solvent used in the Supercrit® process. Furthermore, other results obtained by Depaula et al. demonstrate that the use of hydrogen peroxide at high concentration impacts the biomechanical properties of purified bone matrices, but also the collagen structure which is nevertheless still present in an organized network [31].

In humans, an evaluation of maxillary sinus augmentation using human bone grafts purified with the Supercrit® process showed 10 years after implantations a long-term osseointegration [33]. To minimize the environmental impact of these chemical treatments, a new process, named Goxcrit, was developed, using scCO_2_ and a reduced amount of solvents (i.e., hydrogen peroxide, peracetic acid and ethanol).

Here, structural investigations comparing the “Supercrit®” and the “Goxcrit” processes have shown no differences in the preservation quality of the collagen structure. Nevertheless, a smear layer was observed on the aG samples (SEM images). It is known that the more oxidative and destructive a treatment is, the more the smear layer disappears and the number of micropores increases [34]. These debris stuck in the lacunar and canalicular spaces of the bone confirm the less corrosive effect of this new treatment. They could contribute to the initiation of bone deposition and mediate the connection between the matrix and the newly formed bone observed in our *in vivo* results [35]. This may explain the early appearance in the aG group of an osteoid matrix with its ability to induce increased production of mineralized tissue compared to the other groups. Their presence was not redhibitory, as long as the antigenicity standards were respected, and further characterizations will have to be carried out to meet all the requirements for placing these substitutes on the market as medical devices. In addition, to confirm these results, it would have been interesting to test this process on human bone to see if it would be more effective in preserving the bone matrix of this source. Comparing the decomposition profiles of thermograms obtained with those in the literature, two main phases of degradation in the organic part were observed: one around 337–350 °C, which corresponds to the proteins, and another around 500 °C, which represents the residual proteins [36,37]. The degradation and combustion processes of collagen leads to changes in mass loss between 220 °C and 530 °C [38]. The similarity of thermograms profiles between the scCO_2_, the Supercrit® and Goxcrit processes strongly suggests that collagen was indeed preserved. The spectrum of the remaining organic substances was similar to the FTIR spectrum of collagen that can be found in the literature [39]. Interestingly, a similar FTIR profile was reported in a study investigating collagen preservation in another porcine-derived bone substitute compared to a human-derived one [40]. Similarly, our results show that both processes do not alter the integrity of the bone mineral phase. Nevertheless, additional analyses could be carried out to significantly strengthen our initial conclusions [41].

Nevertheless, to be a viable option, bone xenografts derived from swine must overcome hyperacute rejection, which is mainly caused by the α-Gal antigen [42]. This protein is extensively expressed on the surfaces of porcine bone marrow cells, osteocytes, osteoblasts, and Haversian canals [43]. The persistence of cellular structures in the scCO_2_ condition justified the need to complete the process using chemical compounds, that still remains a certain limitation in the Goxcrit process. Indeed, no histological marking against the α-Gal antigen was present in the samples treated by “Supercrit®” or “Goxcrit”, either human or porcine, showing the efficacy of the two processes.

Due to the use of different chemical compounds, cytotoxicity was assessed. MC3T3 cells were routinely used to test the cytotoxicity of implantable materials for bone regeneration [44,45]. No differences were found in terms of morphology or number of visible cells seeded on our samples with membrane extension profile similar to those of unstressed cells [46].

Although the *in vitro* experiments as a whole showed no significant difference between the different treatments, the *in vivo* results were more discriminating, leading us to hypothesise that the less aggressive procedure should allow better preservation of the bone matrix and result in better cell invasion than the other two groups treated with Supercrit®. The SEM observation of the smear layer, which was not observed with the Supercrit® procedure, supports our conclusions and would favour the formation of osteoid tissue, which is essential for bone remodeling. In the future, it appears relevant to test the Goxcrit process on human bone, as in the present study we focused on porcine samples to be consistent with our research objectives to develop a new xenogeneic substitute at least as effective as the current human Supercrit®.

In the Goxcrit condition, as well as in all other conditions, bone formation was initiated from the edge of the defect where the native bone and the graft were in close contact, as described in physiological bone healing [47]. Whenever mineralization occurred within the implant, the new tissue displayed all the characteristics of a remodeling bone tissue including alternating organized layers and clusters of fibrillar collagen, osteoclast and osteoblast activity, and well-distinguishable vascularization, essential for bone healing [48,49]. This bone remodeling activity was greater in the aG condition, with closure of the lacunae with a fully native bone, showing that implants treated with the “Goxcrit” process could be more rapidly replaced by native bone tissue than in the other conditions. However, in all conditions, most of the mineral structure of the implants was preserved.

Nevertheless, this study was limited by the *in vivo* model used and should be confirmed in large animal models more similar to humans. In addition, the calvaria is a flat bone without mechanical load that have an important function in bone remodeling process [50]. The absence of spongious bone and the poor vascularization of calvaria could also impact the bone regeneration process. Many other variables also need to be studied, such as the impact of these treatments on the substitutes mechanical properties, the influence of female hormones and systemic diseases with repercussions on vascularization and bone functionality [[51], [52], [53]]. In an effort to find a method that does not involve chemical treatments while remaining safe for the patient, studies continue to improve natural bone substitutes. In addition, after purification treatment, the incorporation of biomolecules involved in proliferation and bone remodeling, such as FGF-2 or anti-sclerostin antibody, could be interesting to further enrich these bone substitutes [54,55].

## 5 Conclusion

As a biomaterial innovation, this study associated the Goxcrit process to a xenogeneic porcine bone to address the current demand in regenerative medicine for bone substitutes. Through an optimized supercritical process, Goxcrit preserved the structural integrity of the bone matrix maintaining key biological properties such as porosity and fibrous collagen architecture critical for cellular adhesion and osseointegration. Both *in vitro* and *in vivo* analyses confirmed the absence of toxicity and the alpha-gal immunogenic protein, and an increase in the bone formation. This would suggest that the Goxcrit approach better retains the natural composition and regenerative potential of porcine bone tissue, despite the significant reduction of the solvent used. Nevertheless, this process should then be studied on different allogenic or xenogeneic sources to reach a more general conclusion on its superiority to the patented Supercrit® process.

## CRediT authorship contribution statement

**Solène Rota:** Writing – review & editing, Writing – original draft, Visualization, Validation, Methodology, Investigation, Formal analysis, Data curation, Conceptualization. **Ludovic Sicard:** Writing – review & editing, Writing – original draft, Visualization, Validation, Methodology, Investigation, Formal analysis, Data curation, Conceptualization. **Justine Perarnaud:** Writing – review & editing, Visualization, Investigation, Data curation. **Rémy Agniel:** Writing – review & editing, Visualization, Investigation, Formal analysis. **Raphaël Bardonnet:** Writing – review & editing, Validation, Supervision, Methodology, Conceptualization. **Catherine Chaussain:** Writing – review & editing, Visualization, Validation, Methodology. **Michel Boissière:** Writing – review & editing, Visualization, Validation, Data curation, Conceptualization. **Emmanuel Pauthe:** Writing – review & editing, Visualization, Validation, Supervision, Project administration, Methodology, Funding acquisition, Formal analysis, Data curation, Conceptualization. **Caroline Gorin:** Writing – review & editing, Writing – original draft, Visualization, Validation, Supervision, Methodology, Investigation, Formal analysis, Data curation, Conceptualization.

## Funding

This work was supported by the 10.13039/501100003032ANRT, BIOBank industry, CY university foundation (ReTis Chair) and Université Paris Cité.

## Declaration of competing interest

The authors declare the following financial interests/personal relationships which may be considered as potential competing interests:Solene Rota reports financial support was provided by French National Association of Technical Research. Solène Rota report equipment, drugs, or supplies was provided by BioBank Industry. Solène Rota reports now a relationship with BioBank Industry that includes: employment, she was a PhD student during this work and was then recruited by BioBank industry at the end of her PhD. Raphaël Bardonnet reports a relationship with BioBank industry that includes: board membership and employment. Solène Rota, Raphaël Bardonnet, Michel Boissière, Emmanuel Pauthe have patent pending to PCT/FR2023/051010. If there are other authors, they declare that they have no known competing financial interests or personal relationships that could have appeared to influence the work reported in this paper.

## Figures and Tables

**figs1:**
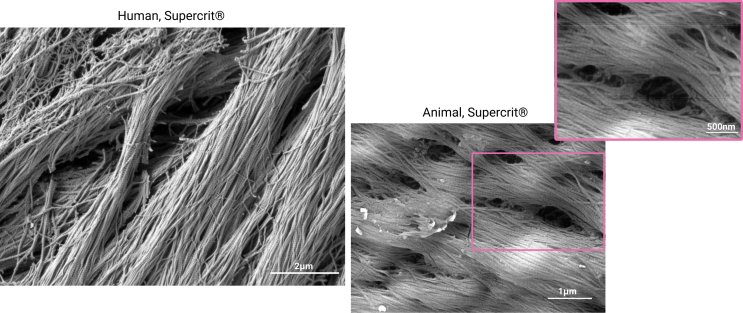
Scanning electonic microscopy of human and animal bone samples after Supercrit® treatment. The fibrils displayed alternating dark and light phases, which are characteristic of collagen fibres, in both human and animal samples, with comparable entanglements. Insight (pink frame) of the animal sample treated with Supercrit® emphasing the arrangement of collagen fibres

**figs2:**
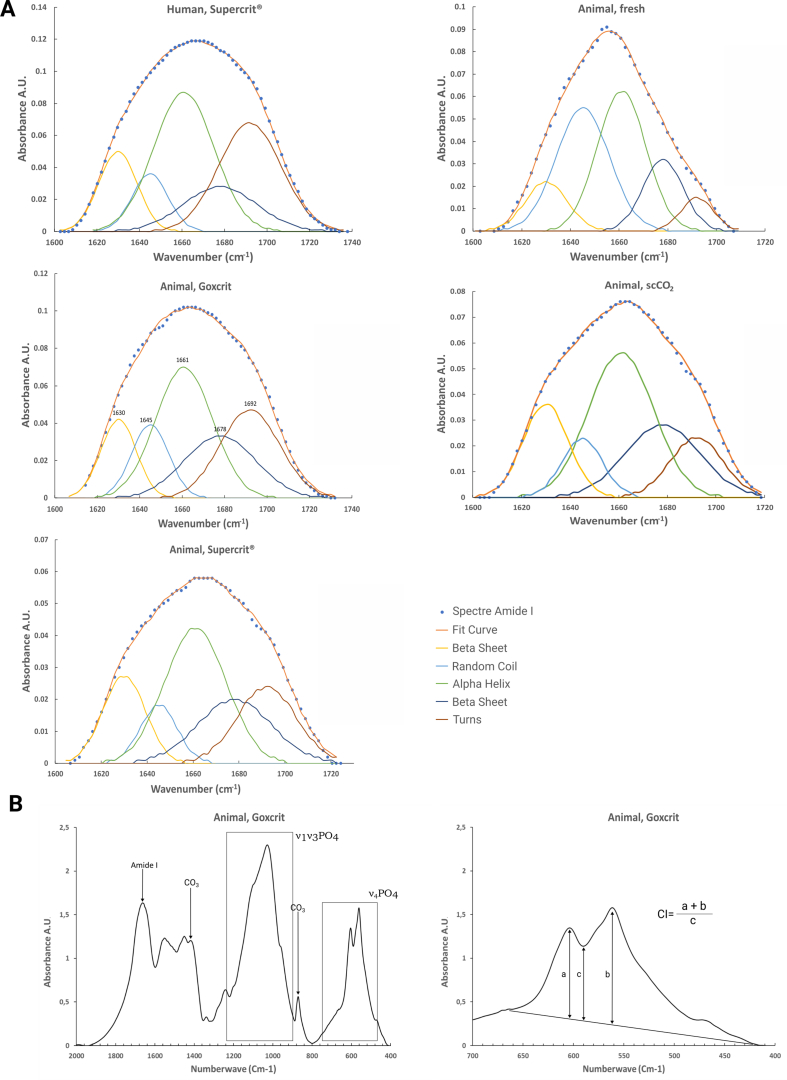
Organic and mineral phase analysis from FTIR Spectra. A: Organic Phase Analysis: Curve-fitting analysis of FTIR Amide I Band of Bone Specimen after different process. The Amide I band (ν(C=O) vibrations) is particularly informative as it reflects the secondary structure of the collagen, especially α-helices, random coils, but also β-sheets, and turns. To quantitatively estimate the relative proportions of these secondary structure components, a curve-fitting treatment was applied. Using the fourth derivative function calculated using Opus software five major components have been identified from the decomposition of the amide I. B: Mineral Phase Analysis: Vibrational groups of the PO4 modes and crystallinity index calculation. The mineral phase in bone is primarily composed of hydroxyapatite, a crystalline structure of calcium phosphate.

**figs3:**
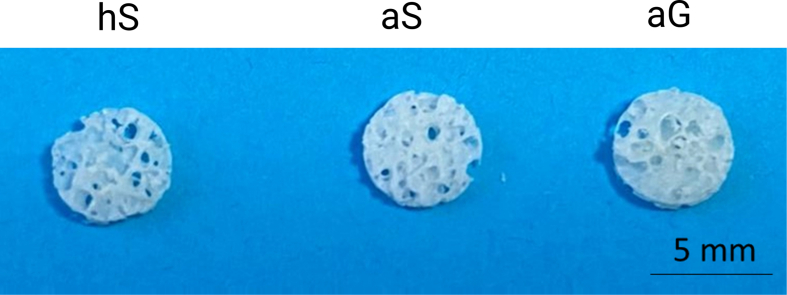
Macroscopic view of the different conditions of the bone implants. hS: human Supercrit®, aS: animal Supercrit®, aG: animal Goxcrit. Scale bar 5mm.

**figs4:**
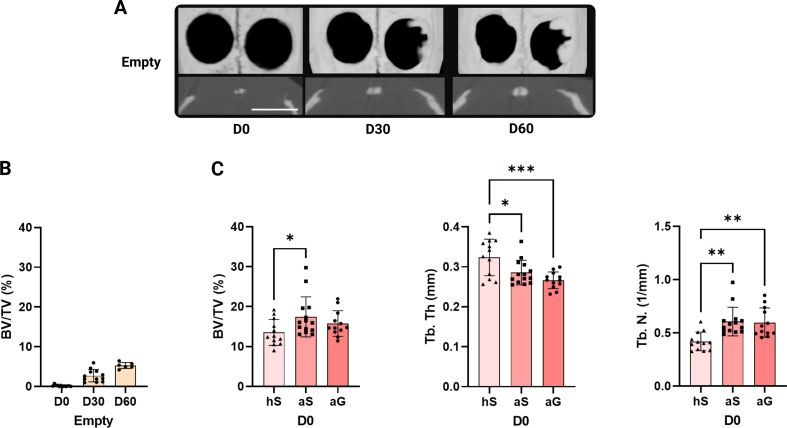
A: Three dimensionnal reconstructions and coronal sections of empty defects at D0, D30 and D60, showing partial reparation from the edges. Scale bar 5mm. B: Bone Volume Tissue Volume ratio (BV/TV) of Empty condition, showing very partial bone reparation over 60 days (0,2±0.2%, 2,7±1.5%, 5,3±0.8% for D0, D30 and D60 respectively). C: BV/TV, Trabecular thickness (Tb. Th) and trabecular number (Tb. N.) of the different conditions at D0. A comparison of the trabecular structure of bone from human (hS) and animal (aS and aG) sources revealed that the former exhibited a reduced number of trabeculae and a greater thickness, but with a smaller volume of bone in the samples. ∗ p<0.5, ∗∗ p<0.01, ∗∗∗ p<0.001.

**figs5:**
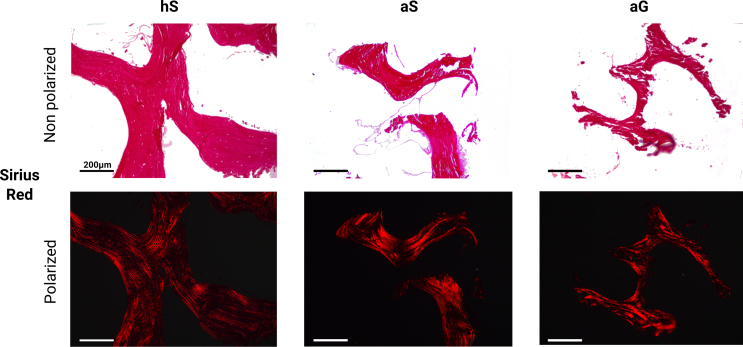
Sirius red staining sections of the non-implanted bone material of the three conditions (hS: human Supercrit®, aS: animal Supercrit®, aG: animal Goxcrit), with non-polarised and polarised light, showing the regular alternation of fibrillar collagens layers (in red) and non fibrillar collagen (in black). Scale bar: 200μm.

## Data Availability

Data will be made available on request.
