# Supplementary material for: Purified bone xenografts: A novel and efficient animal bone substitute derived from an optimized supercritical CO2 treatment
Source: Mater Today Bio. 2025 Mar 3;31:101619. doi: 10.1016/j.mtbio.2025.101619 (PMC11930176; doi:10.1016/j.mtbio.2025.101619)
Supplement: Multimedia component 1 [file mmc1.docx]

**Supplementary tables:** Table corpus about FTIR analysis

| **Bands**  **(cm^-1^)** | **Secondary structure** | **Animal, Fresh** | **Animal, Goxcrit** | **Animal, Supercrit®** | **Animal, CO_2_** | **Human, Supercrit®** |
| --- | --- | --- | --- | --- | --- | --- |
| **1630** | **β−Sheets** | 10.6% | 12.4% | 15.9% | 17.3% | 13% |
| **1645** | **Random Coils** | 33.9% | 12% | 9.2% | 9.7% | 8.8% |
| **1661** | **α−Helix** | 35.15% | 32.9% | 35.5% | 38.3% | 35.6% |
| **1678** | **β−Sheets** | 14.8% | 19.6% | 20.1% | 21.8% | 13.9% |
| **1692** | **Turns** | 5.5% | 23.2% | 19.3% | 12.8% | 28.7% |
| **Cross-links Ratio**  **(1661/1692 cm^-1^)** | | 6.38 | 1.42 | 1.83 | 2.98 | 1.24 |

**Table S1:** Relative area of amide I components (%) and cross-links ratio 1661/1692 cm^-1^. This table summarizes the representative values of our measurements. The "Fresh" animal sample can be considered the most native state of the bone studied. In this case, the Amide I band exhibits two major components at 1645 cm⁻¹ and 1661 cm⁻¹, corresponding to random coil and alpha-helix secondary structures, respectively, as expected and largely described in the literature. Regarding the treated bone samples, regardless of the applied protocol, a clear predominance of the alpha-helix component is observed, while the contribution of random coil structures is significantly reduced. This trend is likely due to the extraction of matrix proteins still present in fresh bone, demonstrating the effectiveness, but also the equivalence of the different treatments.

| **Band Area/Phosphate band Area** | **Animal, Fresh** | **Animal, Goxcrit** | **Animal, Supercrit®** | **Animal, CO_2_** | **Human, Supercrit®** |
| --- | --- | --- | --- | --- | --- |
| **Amide I/ν_1_ν_3_PO_4_** | 0.24 | 0.16 | 0.14 | 0.18 | 0.12 |
| **ν_2_**CO_3_**/ν_1_ν_3_PO_4_** | 0.024 | 0.026 | 0.025 | 0.025 | 0.029 |

**Table S2:** Proportion of Amide I and Carbonate to Phosphate in different samples. This region provides insight into the presence of carbonate within the structure: a shift in the 950 cm⁻¹ ν₁ PO₄ band indicates carbonate loss.

|  | **Animal, Fresh** | **Animal, Goxcrit** | **Animal, Supercrit®** | **Animal, CO_2_** | **Human, Supercrit®** | **Calcined Bone** |
| --- | --- | --- | --- | --- | --- | --- |
| **Crystallinity Index** | 2.22 | 2.52 | 2.55 | 2.49 | 2.60 | 3.16 |

**Table S3:** Value of bone crystallinity Index for each kind of sample. A Crystallinity Index (CI) is calculated by measuring the ratio of absorption peaks. Specifically, the CI was determined by summing the peak heights at 605 cm⁻¹ and 565 cm⁻¹ and dividing this sum by the height of the minimum between these two peaks. The CI is directly related to the organization of the mineral phase, higher values indicate a more structured and crystalline mineral phase.
